# An intron 9 containing splice variant of PAX2

**DOI:** 10.1186/1479-5876-7-36

**Published:** 2009-05-25

**Authors:** Antonia Busse, Anika Rietz, Stefan Schwartz, Eckhard Thiel, Ulrich Keilholz

**Affiliations:** 1 Dept of Medicine III, Charité, Campus Benjamin Franklin, Berlin, Germany

## Abstract

**Background:**

PAX2 is a transcription factor with an important role in embryogenic development. However, PAX2 expression was frequently identified in neoplasia responsible for the growth and survival of cancer cells. Due to alternative splicing of exon 6, exon 10 and exon 12 four isoforms of PAX2 are described so far.

**Methods:**

The expression of an intron 9 containing PAX2 splice variant was analyzed in neoplastic B cell and solid tumor cell lines as well as in primary tumor samples by quantitative RT-PCR. PAX2 proteins were detected by Western Blot in a subset of cell lines.

**Results:**

All 14 lymphoma cell lines expressed an undescribed PAX2 splice variant containing the entire intron 9 sequence and the exon 10 sequence. This splice variant could also be detected in 35 solid tumor cell lines, in leukemia and lymphoma as well as in colon carcinoma and melanoma patient samples and in blood samples of healthy donors. Expression of this new splice variant on protein level was verified by Western Blot analysis.

**Conclusion:**

We discovered a previously undescribed intron 9 and exon 10 containing splice variant of PAX2 in B-cell neoplasia and in solid tumors on mRNA and protein level.

## Background

The PAX gene family was first described in Drosophila and later found to be conserved across species [1]. PAX gene products function as transcription factors. They all share the evolutionarily conserved 128 amino acid paired domain at their N-terminal, which mediates attachment to DNA sequences [2]. Nine PAX genes (PAX1–PAX9) have so far been described in vertebrates; these proteins are subdivided into four classes based on the presence of conserved sequence motifs, the octapeptide (repression domaine) and the homeodomaine (DNA binding domaine) [3]. The PAX2 gene is located on the short arm of chromosome 10, locus 24–25 [4] and encodes a transcription factor that has a critical role in the development of the urogenital tract, the eyes and ears, and the CNS [5]. It belongs to the subgroup 2, characterized by the octapeptide sequence and a truncated homeodomaine [6] and is composed of 12 exons spanning approximately 86 kb [5].

Although PAX2 is primarily expressed during embryonic development and expression is normally repressed upon terminal differentiation, PAX gene expression was frequently identified in tumor cell lines, including lymphoma, breast, ovarian, lung, and colon cancer, as well as in primary tumor tissue samples [7] and was suggested as a sensitive marker for renal neoplasms [8].

Apoptosis was induced in cell lines following RNA interference to silence PAX2 expression, suggesting that endogenous PAX2 gene expression is required for the growth and survival of cancer cells [9,10,7]. Therefore, it has gained interest as a target for immunotherapy [11]. Downstream targets of PAX2 are still less defined. PAX2 has been reported, to act as a transcriptional repressor of p53 and a transcriptional activator of WT1 [12]. In breast [13] and prostate cancers [14] as well as in acute myeloid leukemia (AML) [15] a correlation with WT1 expression has been observed, suggesting that PAX2 is a positive transcriptional regulator of WT1. Recently, WNT5A [16] and human beta-defensin-1 [17] were identified as PAX2 targets.

Four isoforms of PAX2 are described so far. They are products of alternative splicing of exon 6, exon 10 and exon 12: Exon 6 is present in the PAX2a transcript and absent in the PAX2b transcript [18]. Insertion of exon 10 in the exon 6 missing PAX2c transcript results in a different reading frame, and a stop codon is produced by the last three bases of exon 11 [19]. PAX2d arises from deletion of the first 19 bp of exon 12 and is found with and without exon 6 (PAX2d+ex6 und PAX2dΔex6) [20].

Here we characterized a previously undescribed intron 9 and exon10 containing splice variant of PAX2 in neoplastic B cell lines and solid tumor cell lines as well as in tumor tissue.

## Methods

### Cells and Reagents

14 lymphoma cell lines (AMO-1, DG75, EHEB, KARPAS-422, KM-H2, HDLM-2, L540, RAJI, SU-DHL-4, SUP-M2, U698, U937, U266, BONNA-12) and 35 solid tumor cell lines (4 thyroid cancer cell lines: 8505C, CGTHW-1, BCPAP, TT260Co2; 7 renal cell carcinoma cell lines: A706; Caki1; ACHN; A498; SN12; CC5; Caki2; 8 melanoma cell lines: SKMel23, Mel10, Mel16, Mel-HO, SKMel24, SKMel5, Mel28, 624.28; 8 colon carcinoma cell lines: SW620, HCT116, Cx94, CaCo2, Colo320, SW480, Colo205, HBL 100, 5 breast cancer cell lines: Mx1, T47D, MCF7, MDA-MB436, BT474; 3 lung carcinoma cell lines: Column6, A427, DMS79) All human cell lines were purchased from DSMZ (Braunschweig, Germany) and CLS (Eppelheim, Germany). Cells were maintained in RPMI 1640 containing 10–20% FCS, 2% penicillin/streptomycin and 2% glutamine (Gibco, Karlsruhe, Germany).

### Patient samples

Fifteen primary low grade lymphoma, 9 myeloma, 11 acute lymphoblastic leukemia (ALL) samples and 7 AML samples were taken from patients that underwent routine diagnostics like venipuncture or bone marrow aspiration. Primary tumor single cell suspensions were prepared by ficoll hypaque separation. The lymphoma and leukemia samples contained more than 80% of tumor cells; therefore no further separation was done. For multiple myeloma, CD138 positive cells were isolated using Mini MACS technology (Miltenyi Biotec, Germany). Tumor cells were resuspended in guanidium thiocyanate (GTC) buffer and stored at -80°C. 12 melanoma (8 skin melanoma, 4 ocular melanoma) and 12 colon carcinoma tissue samples were obtained from patients that underwent surgery for their tumors. Tissue samples were collected and dissected under stringent sterile conditions to prevent RNA contamination and immediately frozen in liquid nitrogen. There were no specific inclusion criteria with exception for the leukemia samples. Only PAX2 mRNA expressing AML and ALL samples were included. All patients had given informed consent for the analysis. Approval by the appropriate ethics committee has been obtained (approval number EA4/090/08) and analyses have therefore been performed in accordance with the ethical standards laid down in the 1964 Declaration of Helsinki. Blood samples of healthy volunteers served as negative controls.

### RT-PCR

Total RNA was isolated by RNeasy Mini Kit including RNase-Free DNase Set (Qiagen, Hilden, Germany). Reverse transcription and quantitative Real Time RT-PCR (LightCycler Technology, Roche Diagnostics) was done as described elsewhere [15]. Primer sequences were designed using the LightCycler Probe Design software, version 1.0 (LC, LightCycler; P, dephosphorylated; X, Fluorescein; Y, LC Red 640): PBGD Forward: 5'-TGC AGG CTA CCA TCC ATG TCC CTG C, Reverse: 5'-AGC TGC CGT GCA ACA TCC AGG ATG G, LC probes: 5'-Y TGT GGG TCA TCC TCA GGG CCA TCT TC P, 5'-CGT GGA ATG TTA CGA GCA GTG ATG CCT ACC X, 187 bp. PAX2_1 Forward: 5'-CTGGTCGTGACATGGC, Reverse: 5'-GGGTTGCACACAAGGG, LC probes: 5'-Y ACCCTGGCAGGAATGGT P, 5'-GGGAAGCTACCCCACCT X, 185 bp; PAX2_2 Forward: 5'-GGTTACCCCCCTCACG, Reverse: 5'-GGGACAGAATAGCAGTGG, LC probes: 5'-Y GGTGCCTGGTAGGTGACAA P, 5'-CCTCCACCCTGGCAGGA X, 212 bp.

PCR conditions and target-specific final MgCl_2 _concentrations are listed in table 1. For each target an initial denaturation cycle at 95°C for 10 min, a final extension cycle at 72°C for 2 min was performed. For quantification, PCR products were cloned into the vector pCR2.1-TOPO (Invitrogen, Groningen, The Netherlands). A standard curve with 3 dilutions of the appropriate plasmid in duplicates was included in each PCR run. The specificity of the PCR products was confirmed by melting curve analysis, by gel electrophoresis using the AlphaEaseFC Imaging software (Alpha Innotech, San Leandro, CA) and by sequencing.

**Table 1:** PCR conditions and specific MgCl_2 _concentrations for the amplification of PAX2 transcripts and the housekeeping gene PBGD.

PCR conditions				
target	MgCl2 (mmol/l)	Cycles	Temperature (°C)	Time (s)
PBGD	4	45	95	0
			65	12
			72	10
PAX2_1	1,5	55	95	0
			55	10
			72	8
PAX2_2	2	55	95	0
			57	12
			72	10

### Data analysis/statistical analysis

Analysis of RT-PCR expression data was done with the LightCycler software (version 3). Sample concentrations were calculated using the plasmid standard curve resulting in marker concentrations. All samples were analysed in duplicate. The average value of both duplicates was used as a quantitative value. To correct for differences of cDNA amount on a per-sample basis, results were provided as ratio to housekeeping gene porphobilinogen deaminase (PBGD) expression. Statistical significance was tested using SPSS 15.0 software. For comparison of PAX2 intron 9 specific mRNA expression levels significance was estimated by the 2-sided Mann-Whitney U test for comparison of two different groups.

### Detection of PAX2 proteins by Western Blot

Western blots were performed on equal amounts of protein obtained by lysis of cells using MPer Protein Extraction Reagent (Pierce, Rockford, USA). The protein concentration was measured by BCA method using BCA Protein Reagent (Pierce, Rockford, USA). 50 μg protein extract was loaded onto a 10% SDS-PAGE (Pierce, Rockford, USA). Following electrophoreses, proteins were transferred to nitrocellulose membranes, and then blocked with 1%BSA in PBST (1× PBS, 0.1% Tween) overnight at 4°C. Blots were then probed with rabbit anti-PAX2 primary antibody (Zymed, San Francisco, USA) at 1:1000 dilution. After washing with PBST the membranes were incubated with anti-rabbit IgG antibody conjugated to horseradish peroxidase (HRP) at 1:5000 dilution (Amersham, UK). Signal detection was visualized using ECL chemiluminescence reagent (SuperSignal West Dura Trial Kit, Pierce, Rockford, USA). As a control, blots were probed with mouse anti-β-actin primary antibody (1:2000, Sigma, Deisenkirchen, Germany) and HRP-conjugated anti-rabbit secondary antibody.

## Results and discussion

### Detection of a new splice variant in tumor cell lines and tissue by RT-PCR

**Figure 1:**
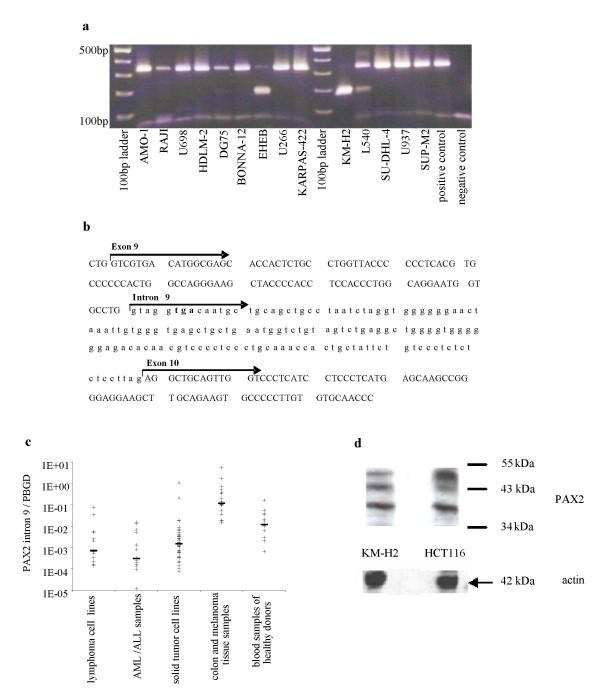
**A: Agarose gel electrophoresis of the PAX2 exon 10 RT-PCR products from the mRNA of the lymphoma cell lines**. All lymphoma cell lines: band of 339 bp of varying intensity. KM-H2, EHEB, L540 and DG75: band of the expected size of 185 bp. Negative control: water instead of cDNA, positive control: plasmid (pCR2.1-TOPO) coding for the PAX2 exon 10 PCR product. **B: Schematic presentation of the sequencing result of the 339 bp PCR product**: Detection of the new PAX2 splice variant containing the whole intron 9 sequence and exon 10 sequence **C: Expression level of PAX2 intron 9 specific mRNA**: The relative amount was expressed as ratio marker [pg/μl]/PBGD [pg/μl]). The sample concentration was calculated using the plasmid standard curve. Thick bar: median expression level. **D: Analysis of the expression of the different PAX2 splice variants by Western Blot**: The known splice variants of 43–46 kDa and the new splice variant of 37 kDa are exemplarily shown for the colon carcinoma cell line HCT116 and lymphoma cell line KM-H2.

RT-PCR analysis of the PAX2 transcript in 14 lymphoma cell lines using a forward primer lying in exon 9 and a reverse primer lying in exon 10 (primer set PAX2_1) showed different PCR products on gel electrophoresis (figure 1a):

All lymphoma cell lines showed a band of 339 bp of varying intensity. A band of the expected size of 185 bp was detected only in the cell lines KM-H2, EHEB, L540 and to a lesser extent in the cell line DG75. Sequencing analysis of the 339 bp PCR products revealed that this product results from the insertion of the entire intron 9 sequence. Thus, these cell lines expressed an undescribed PAX2 splice variant containing the entire intron 9 sequence and the exon 10 sequence (figure 1b) with a stop codon at the beginning of intron 9.

To analyze, whether the new splice variant is also expressed in solid tumors, a panel of solid tumor cell lines was tested by RT-PCR with the same primer set spanning the intron 9 (PAX2_1). Analysis of the product size by gel electrophoresis showed, that 7 of the 8 melanoma cell lines, 7 of the 9 colon carcinoma cell lines and 1 of the 7 renal carcinoma cell lines expressed the intron 9 and exon 10 containing splice variant. The other cell lines showed only a band of 185 bp. Additionally, 5 of 5 breast carcinoma cell lines, 3 of 3 lung carcinoma cell lines and 4 of 4 thyroid carcinoma cell lines expressed this splice variant.

Next PAX2 positive leukemia patient samples were analyzed: In all 11 ALL samples and all 7 AML samples the new splice variant could be detected on gel electrophoresis. Subsequently, samples from patients with low grade lymphoma and multiple myeloma were analyzed. All 15 low grade lymphoma patient samples and 7 of the 9 multiple myeloma patient samples expressed the intron 9 containing splice variant. The remaining 2 multiple myeloma samples were negative for PAX2 mRNA (determined by an RT-PCR assay detecting all splice variants of PAX2, data not shown). However, 22 of 24 blood samples from healthy donors surprisingly were also positive for the intron 9 and exon 10 containing splice variant.

As PAX 2 recently gained importance as an immunotherapeutic target [11], differences in the quantitative expression levels of this intron 9 positive PAX2 splice variant between tumor cell lines and tissue compared to blood samples from healthy donors were analyzed. A new RT-PCR with intron 9 specific primers (primer set PAX2_2) was established (figure 1c). In all 14 lymphoma cell lines intron 9 specific mRNA could be detected, also in the cell line KM-H2. The median expression level was 7.16 × 10^-4 ^(range 1.42 × 10^-4 ^- 7.61 × 10^-2^). Additionally, in all solid tumor cell lines intron 9 specific mRNA was detected. The median expression was 1.49 × 10^-3 ^(7.96 × 10^-5 ^- 1.04). Moreover, the expression of the intron 9 positive PAX2 isoform was analyzed in 12 melanoma (8 skin melanoma, 4 ocular melanoma) and 12 colon carcinoma patient samples as well as in 9 AML and 5 ALL patients. All leukemia samples, 11 of the 12 colon carcinoma and 7 of the 12 melanoma samples were positive for expression of intron 9 specific mRNA. The median expression level in solid tumor samples was 1.17 × 10^-1 ^(range 1.43 × 10^-2 ^- 5.44) and in leukemia samples 3.07 × 10^-4 ^(range 1.22 × 10^-5 ^- 1.3 × 10^-2^) (figure 1c). The expression level in solid tumor tissue was 2 logs above the expression level of solid tumor cell lines. The difference in PAX2 expression between solid tumor cell lines and solid tumor samples may be due to in-vitro selection in cell lines or stroma cell contribution in tumor tissue.

However, the intron 9 specific mRNA was also found in 13 of 13 blood samples of healthy donors with a median expression level of 1.18 × 10^-2 ^(range 6.33 × 10^-4 ^- 1.63 × 10^-1^) (figure 1c). The median expression level was significantly higher compared to solid tumor cell lines (p = 0.001) as well as lymphoma cell lines (p = 0.004) and leukemia samples (p = 0.001). In contrast solid tumor tissue samples exhibited a significant higher expression level than healthy controls (p < 0.001). However, regarding immunotherapeutic strategies we cannot exclude significant expression of PAX2 intron 9 protein in peripheral blood of healthy subjects and differences in mRNA expression levels may not automatically lead to significant differences in protein expression.

### Detection of the new splice variant by Western Blot

To verify the expression of this intron 9 positive splice variant on protein level, PAX2 protein expression was examined by Western Blot analysis in whole cell extracts of 3 colon carcinoma cell lines (HCT116, Colo320, Caco2) and 4 lymphoma cell lines (SU-DHL-4, KARPAS-422, U266, KM-H2). Protein bands corresponding to known PAX2 isoforms (PAX2a 44.5 kDa, PAX2b 42 kDa, PAX2c 41.8 kDa, PAX2d 43.6 kDa, PAX2e 46.2 kDa) could be found in all cell lines. Additionally, a band of approximately 37 kDa (figure 1d) was identified in all 4 lymphoma cell lines and in 2/3 colon carcinoma cell lines (Caco2, HCT116), which corresponds to size of the new intron 9 and exon 10 containing splice variant. Actin control staining revealed a band of the expected size of 42 kDa in both cell lines.

However, in blood samples of healthy volunteers bands corresponding to the known splice variants of PAX2 and to a lesser extent to the new splice variant could be also detected. Expression of PAX2 in lymphoid cells was also observed by others [8].

Therefore, regarding PAX2 targeted therapies like vaccination strategies caution is needed.

## Conclusion

We found a previously undescribed intron 9 and exon 10 splice variant of PAX2 on mRNA and protein level in B cell neoplasia and solid tumors as well as in peripheral blood of healthy patients. This splice variant has a distinct and a shorter C-terminus than the known exon 10 containing splice variant PAX2c due to the deletion of the last 89 amino acid residues. Alternative processing represents an important mechanism for the generation of various protein isoforms with different functions from one genetic locus [21]. The function of this intron 9 containing splice variant of PAX2 remains unclear, however, as the transactivation of PAX2 relies on multiple COOH-terminal domains [22], one might speculate, that the shortened new splice variant has a reduced transactivation activity.

## Competing interests

Financial Disclosure: Ulrich Keilholz is holding a patent for the use of PAX2 for cancer immunotherapy. All other authors have declared there are no financial conflicts of interest in regards to this work.

Grant Support: EU Integrated Project Cancer Immunology and Immunotherapy, project: WP 02.03 Transcription factors PAX2 and PAX8 as new tumor antigens.

## Authors' contributions

AB has made substantial contributions to conception and design, acquisition of data, analysis and interpretation of data and wrote the manuscript; AR: has made substantial contributions to conception and design, acquisition of data, analysis and interpretation of data. SS have been involved in acquisition of data and revising the manuscript critically for important intellectual content; ET has made substantial contributions to conception and design and was involved in revising the manuscript critically for important intellectual content, UK: has made substantial contributions to conception and design, as well as analysis and interpretation of data and wrote the manuscript.
